# Quadrivalvular Carcinoid Heart Disease Via Patent Foramen Ovale as Initial Presentation of Metastatic Neuroendocrine Tumor

**DOI:** 10.1016/j.jaccas.2026.109223

**Published:** 2026-07-09

**Authors:** Riya Sam, Anil Ananthaneni, Sadichhya Karki, Kevin Lee, Valluvan Jeevanandam, Blase Polite, David M. Najman

**Affiliations:** a Division of Cardiology, Endeavor Health, Glenview, Illinois, USA; b Division of Cardiology, University of Chicago Pritzker School of Medicine, Chicago, Illinois, USA; c Division of Hematology and Oncology, Louisiana State University Health Sciences, Shreveport, Louisiana, USA; d Division of Cardiothoracic Surgery, University of Chicago Pritzker School of Medicine, Chicago, Illinois, USA; e Division of Hematology and Oncology, University of Chicago Pritzker School of Medicine, Chicago, Illinois, USA

**Keywords:** carcinoid heart disease, left-sided valvular involvement, neuroendocrine tumor, NET, patent foramen ovale, quadrivalvular regurgitation

## Abstract

**Background:**

Carcinoid heart disease typically affects right-sided valves. Quadrivalvular involvement is rare.

**Case Summary:**

A 52-year-old woman presented with florid heart failure. Multimodality imaging demonstrated severe multivalvular regurgitation, and a large patent foramen ovale. She underwent bioprosthetic triple valve replacement with atrial septal defect patch closure. Histology revealed fibromyxoid degeneration and postoperative evaluation confirmed a metastatic small bowel neuroendocrine tumor. Following somatostatin analog therapy as a bridge, she underwent definitive oncologic resection with complete biochemical and radiologic response.

**Discussion:**

This case illustrates the indolent course of carcinoid syndrome and the dramatic presentation of severe undiagnosed carcinoid heart disease. Atypical multivalvular disease should prompt carcinoid screening, as preoperative recognition is essential to avoid life-threatening carcinoid crisis.

**Take-Home Messages:**

In patients with unexplained multivalvular regurgitation and suspected neuroendocrine tumor, evaluate for right-to-left shunting and carcinoid valvulopathy to enable timely, multidisciplinary management.

## Visual Summary

**Figure:**
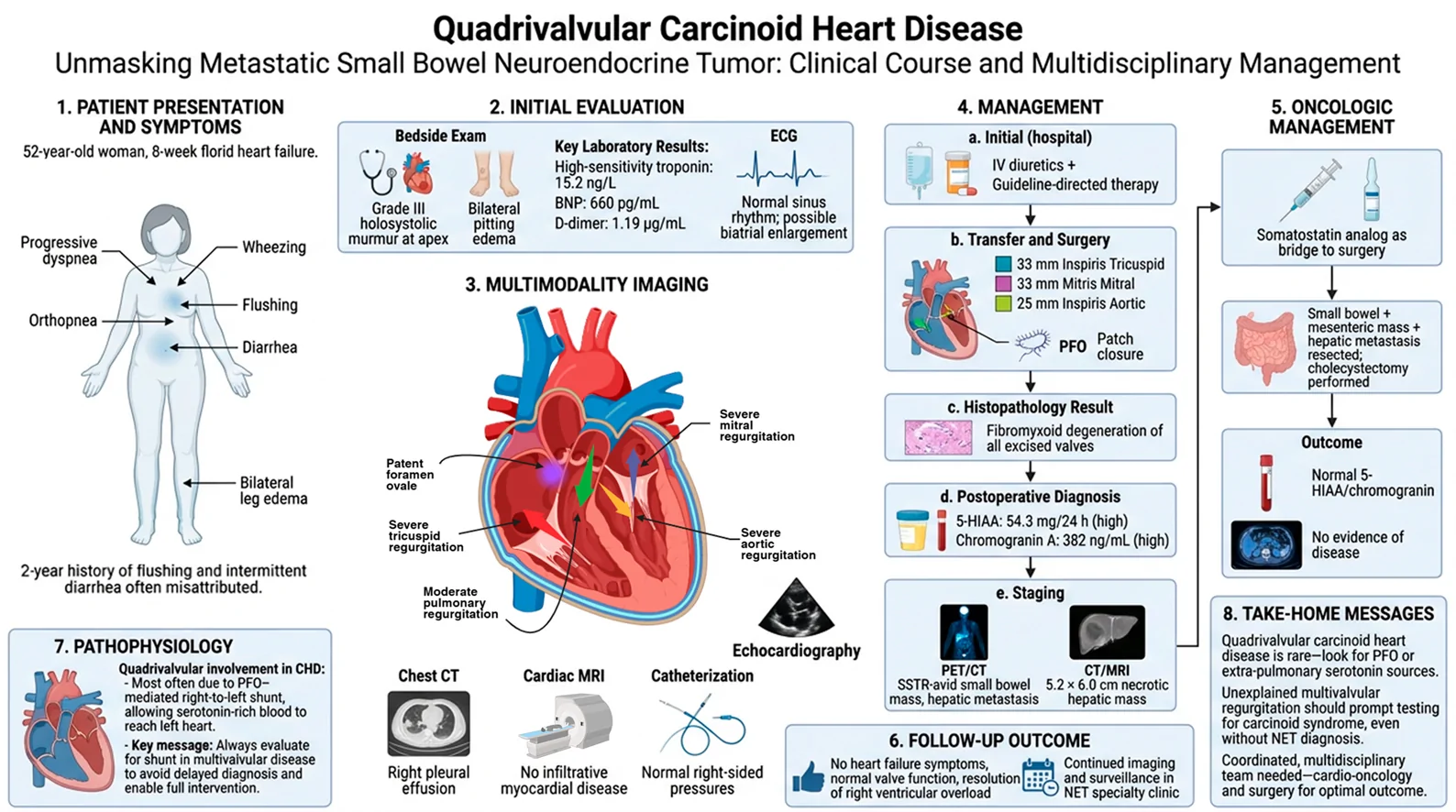

**Visual Summary:**
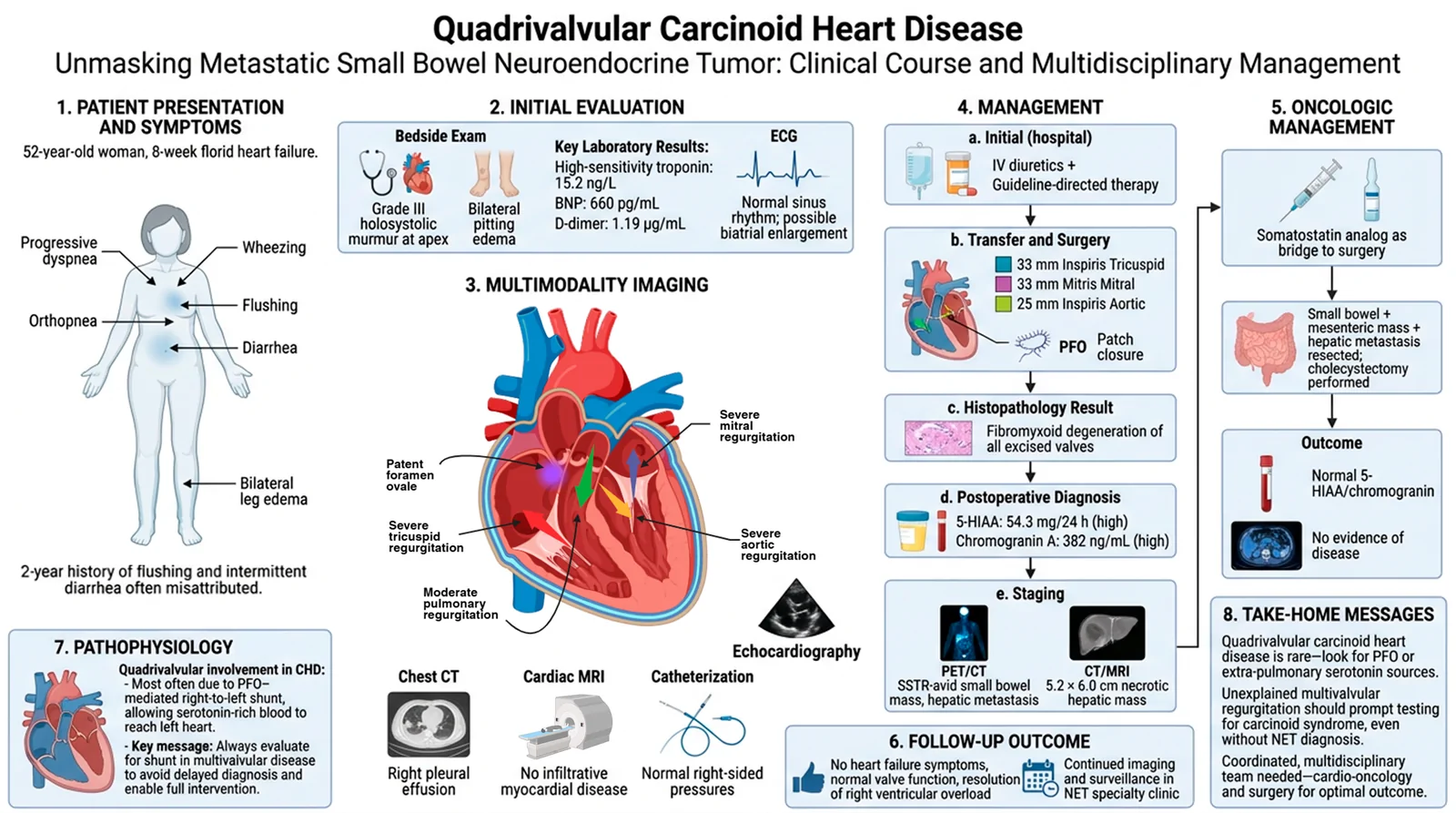
Quadrivalvular Carcinoid Heart Disease Mediated By Patent Foramen Ovale Clinical Course, Management, and Oncologic Outcomes in a 52-Year-Old Woman With Occult Metastatic Neuroendocrine Tumor BNP = B-type natriuretic peptide; CHD = carcinoid heart disease; CT = computed tomography; ECG = electrocardiogram; IV = intravenous; MRI = magnetic resonance imaging; NET = neuroendocrine tumor; PET = positron emission tomography; PFO = patent foramen ovale; 5-HIAA = 5-hydroxyindoleacetic acid.

## History of Presenting Illness

A 52-year-old woman presented to an outside hospital with an 8-week history of progressively worsening dyspnea, orthopnea, and bilateral lower extremity edema. A 2-year history of cutaneous flushing had been attributed to perimenopause and subsequent episodes of wheezing, diarrhea, and dyspnea had been ascribed to a presumed milk allergy. On admission, physical examination revealed a grade III holosystolic murmur at the apex, decreased air entry on the right side with bilateral basal crackles, and bilateral pitting pedal edema.

## Past Medical History

Her past medical history included attention-deficit/hyperactivity disorder, anxiety, hyperlipidemia, asthma, migraines, and obstructive sleep apnea. There was no family history of cardiomyopathy or valvular disease.

## Differential Diagnosis

The differential diagnosis at the time of admission was acute decompensated heart failure, valvular cardiomyopathy, and rheumatic heart disease.

## Investigations

**Figure 1:**
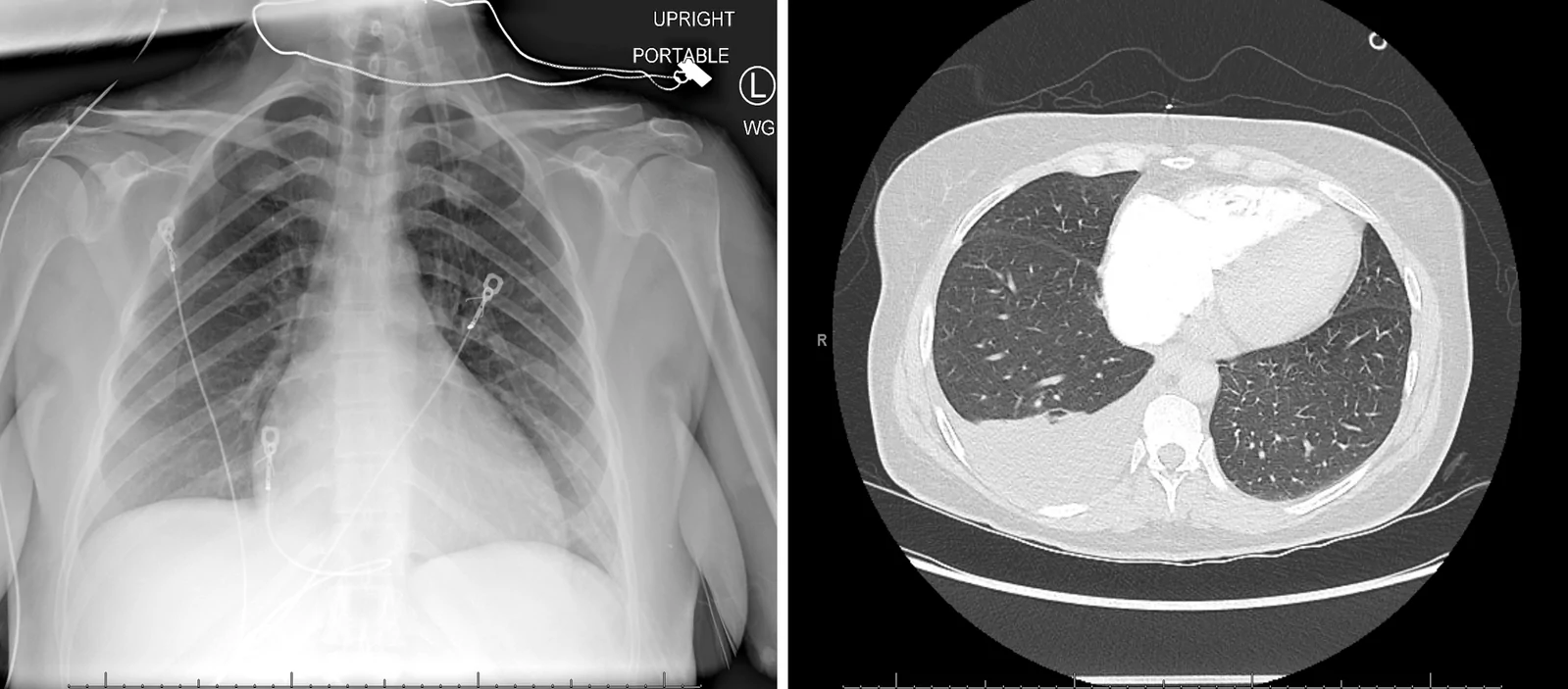
Chest X-Ray With Small Right-Sided Pleural Effusion Computed tomography pulmonary embolism with large right-sided effusion not seen in chest x-ray and minimal left-sided effusion.

Initial laboratory evaluation showed a high-sensitivity troponin of 15.2 ng/L (reference <12 ng/L), B-type natriuretic peptide of 660 pg/mL (reference <100 pg/mL), and D-dimer of 1.19 μg/mL Fibrinogen Equivalent Units (reference <0.5 μg/mL). Electrocardiography demonstrated a normal sinus rhythm with possible biatrial enlargement and nonspecific ST-T wave changes. Chest computed tomography (CT) excluded pulmonary embolism but revealed a large right and a minimal left pleural effusion (Figure 1).

**Figure 2:**
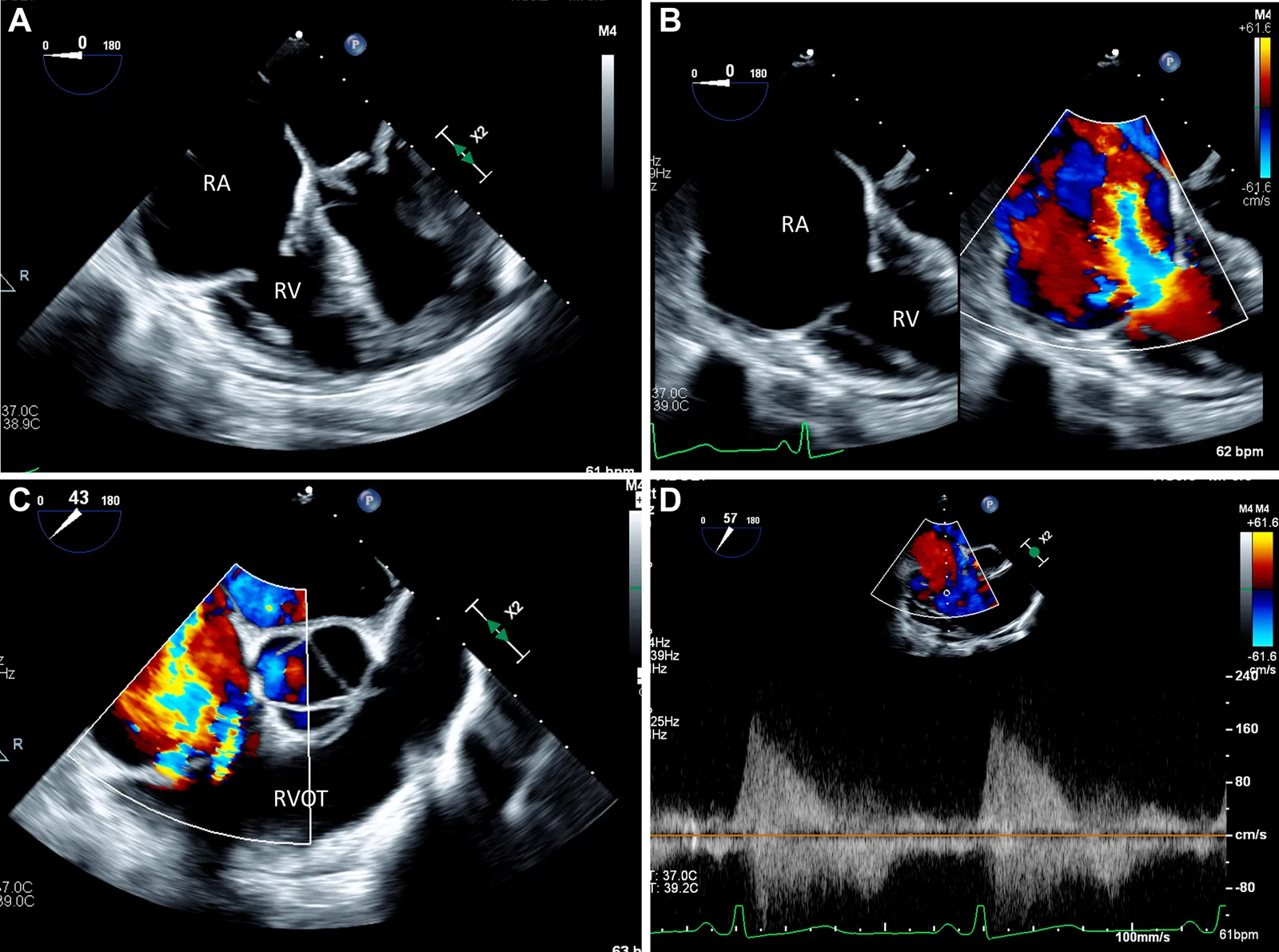
Transesophageal Echocardiography Showing Thickened and Malcoapted Tricuspid Valve With Severe Regurgitation Transesophageal echocardiography midesophageal view showing thickened, retracted, and severe malcoaptation of tricuspid valve (A) with resultant severe tricuspid regurgitation (B, C). Continuous-wave Doppler with dense triangular shape jet with rapid systolic decline characteristic of severe tricuspid regurgitation (D). RA = right atrium; RV = right ventricle; RVOT = right ventricular outflow tract.

**Figure 3:**
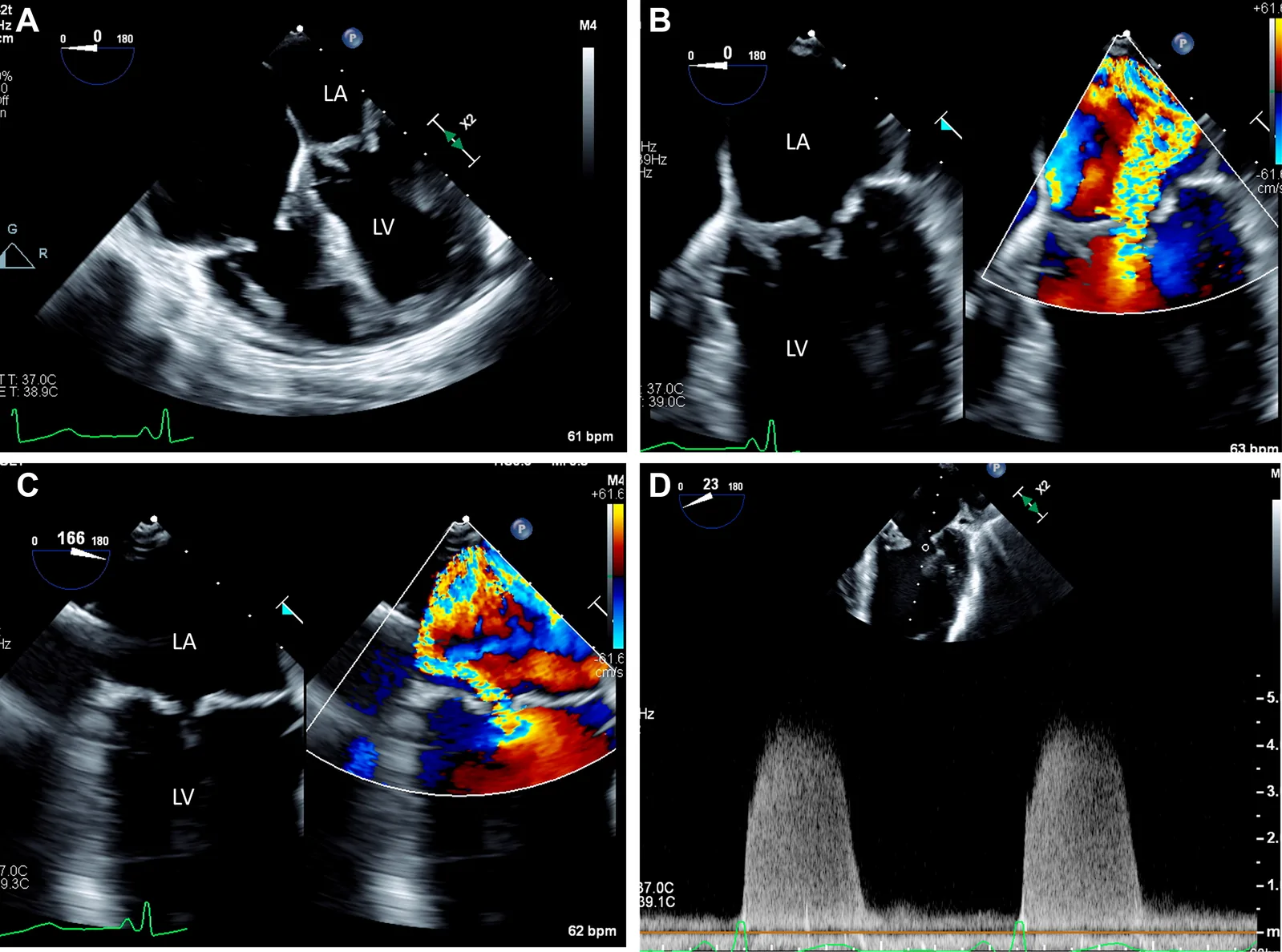
Transesophageal Echocardiography Showing Restricted Mitral Valve With Severe Regurgitation Transesophageal echocardiography midesophageal view showing thickened, mitral valve restricted posterior leaflet and prolapse of the anterior leaflet (A) with resultant severe posteriorly directed mitral regurgitation (B, C). Continuous-wave Doppler with dense jet suggestive of severe MR (D). Abbreviations as in Figure 2.

**Figure 4:**
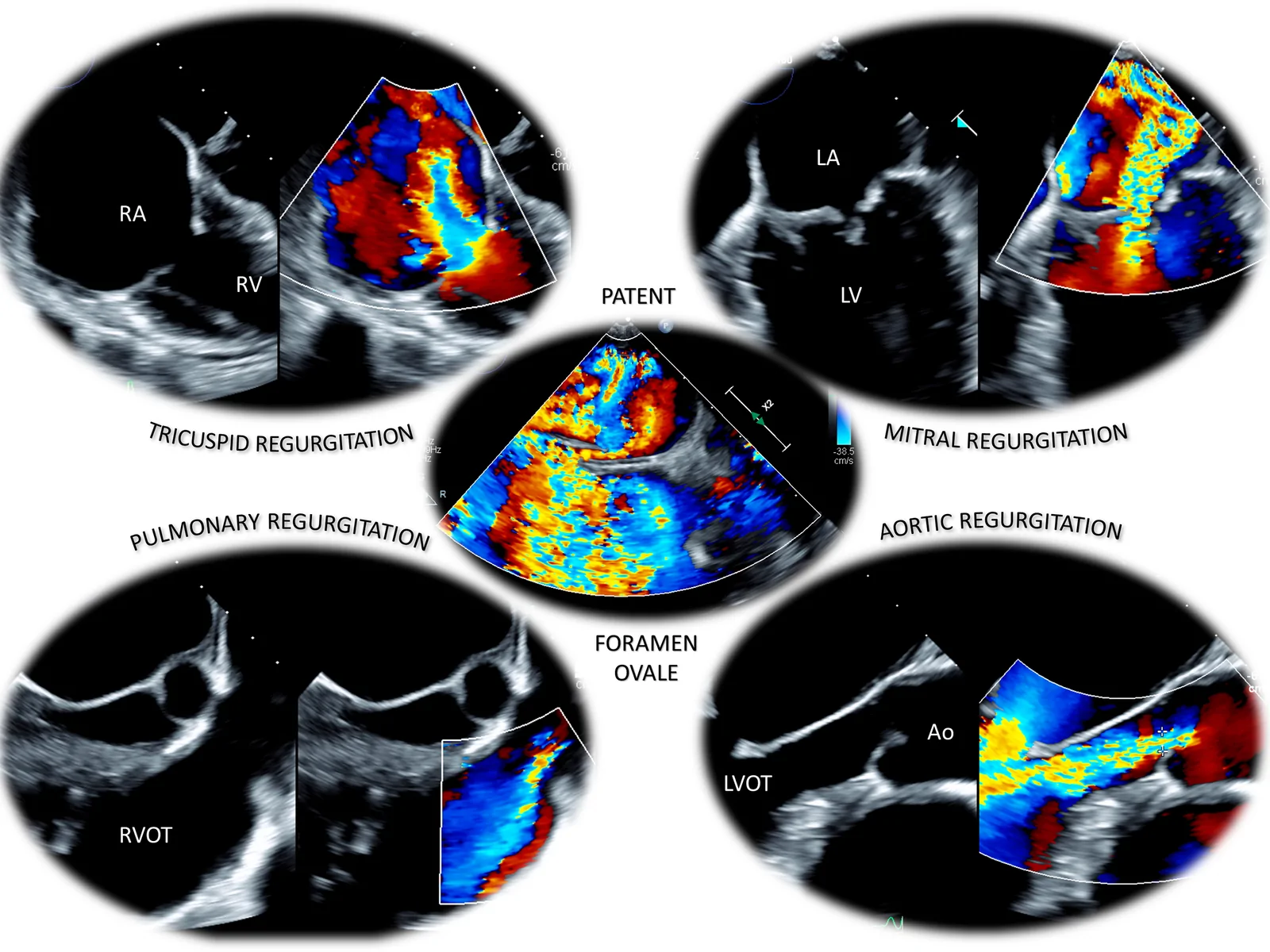
Transesophageal Echocardiography Images Showing Severe Regurgitation of All 4 Valves and a Bidirectional Patent Foramen Ovale Ao = aorta; LVOT = left ventricular outflow tract; other abbreviations as in Figure 2.

Transthoracic echocardiography showed preserved biventricular systolic function, severe biatrial enlargement, and severe tricuspid regurgitation with systolic flow reversal in the hepatic veins. The mitral valve was thickened with anterior mitral leaflet prolapse and resultant severe posteriorly directed mitral regurgitation (Video 1). The aortic valve was trileaflet with severe central aortic regurgitation, no aortic stenosis with a Vmax of 1.7 m/s, aortic valve area by velocity time integral 2.2 cm^2^, mean gradient of 7 mm Hg, and aortic valve dimensionless index of 0.86. In addition, there was mild pulmonary regurgitation (PR) and diastolic septal flattening consistent with right ventricular volume overload. Transesophageal echocardiography confirmed retracted and malcoapting tricuspid leaflets with severe tricuspid regurgitation with dense triangular continuous-wave Doppler jet, Vmax of 1.8 m/s, vena contracta 0.8 cm, and effective regurgitant orifice area 19.4 cm^2^ (Figure 2, Video 2). The mitral valve was thickened and restricted with anterior leaflet prolapse and severe posteriorly directed mitral regurgitation with Coanda effect, effective regurgitant orifice area 0.6 cm^2^, regurgitant volume 105 mL, and mean transmitral gradient 2 mm Hg at a heart rate of 60 beats/min (Figure 3, Video 3). The aortic valve was trileaflet, a small central malcoaptation with severe aortic regurgitation with vena contracta 0.7 cm, Doppler measurements were not obtained (Video 4). The pulmonary valve was normal appearing with moderate PR (Video 5) and a large patent foramen ovale (PFO) with bidirectional shunting on color Doppler (Figure 4, Video 6).There was no stenosis of any of the valves. Subsequent right heart catheterization demonstrated normal right-sided pressures and pulmonary capillary wedge pressure after adequate diuresis. Coronary angiography revealed mild nonobstructive coronary artery disease with a normal left ventricular end-diastolic pressure. Cardiac magnetic resonance imaging confirmed multivalvular regurgitation without infiltrative myocardial disease. PFO was visualized but Qp/Qs could not be calculated because of suboptimal flow measurements. Her mitral valvular pathology was attributed to rheumatic heart disease at this time.

## Management

**Figure 5:**
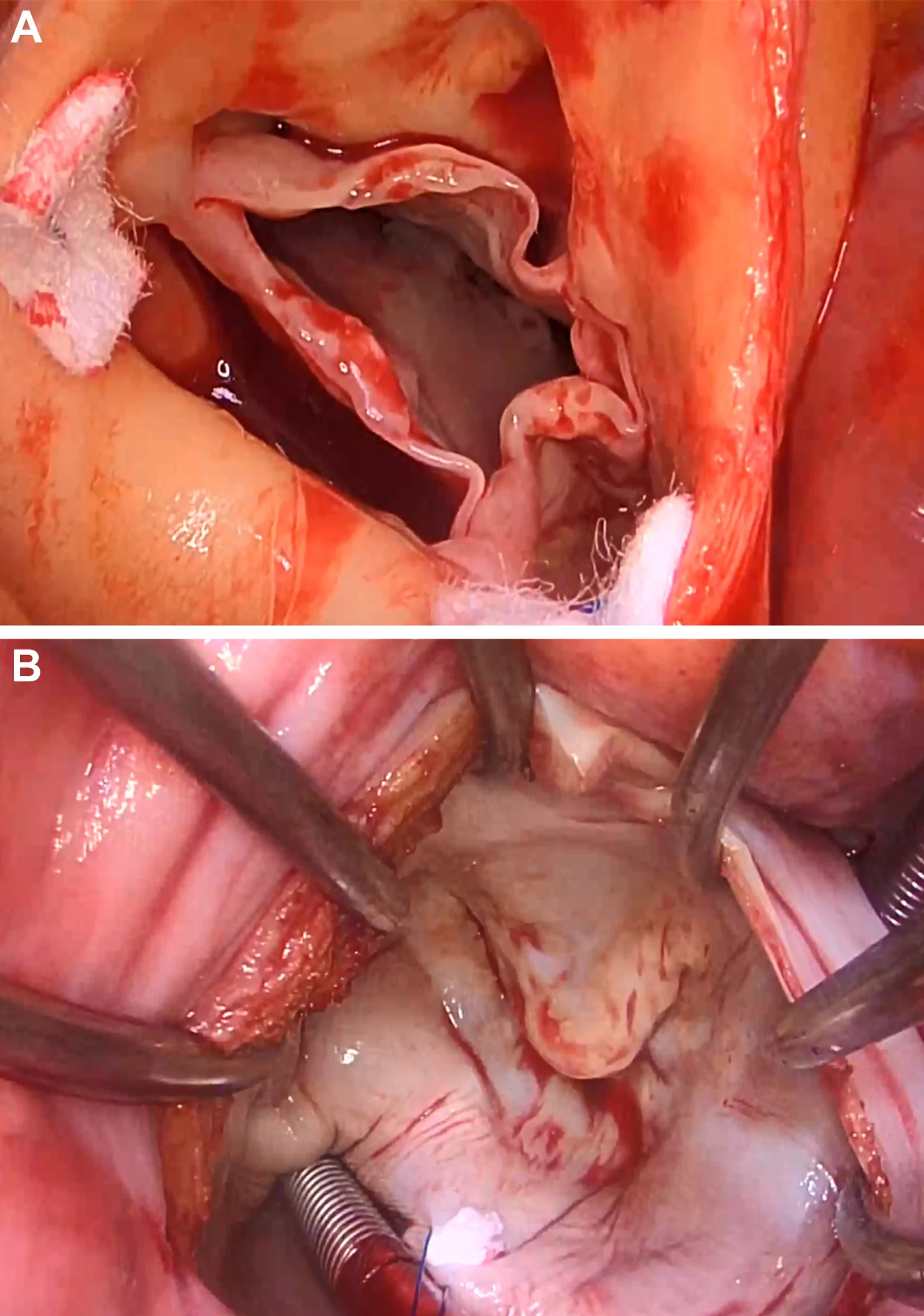
Intra Operative Findings During Triple Valve Replacement Surgery Intraoperative findings (A) thickened, fibrotic, and noncoapted tricuspid valve. (B) Thickened and fibrotic mitral valve with anterior leaflet prolapse.

Intravenous diuretics and low-dose guideline-directed medical therapy produced symptomatic improvement. She was referred to cardiothoracic surgery at a high-volume center and was planned for triple valve replacement as an outpatient. She presented soon after with acute hypoxic respiratory failure requiring noninvasive ventilation. The family sought a second opinion from another cardiologist who, on review of images, recommended evaluation of carcinoid heart disease. She was transferred for an urgent surgical valve replacement and underwent replacement of the tricuspid, mitral, and aortic valves with 33-mm Inspiris, 33-mm Mitris, and 25-mm Inspiris bioprosthetic valves, respectively, along with patch closure of the interatrial septal defect and left atrial appendage ligation. Intraoperative findings confirmed a thickened and malcoapted tricuspid valve as well as a restricted and fibrotic mitral valve with subvalvular fibrosis (Figure 5). She experienced no hemodynamic instability perioperatively. Histopathologic examination of all excised valves demonstrated fibromyxoid degeneration.

**Figure 6:**
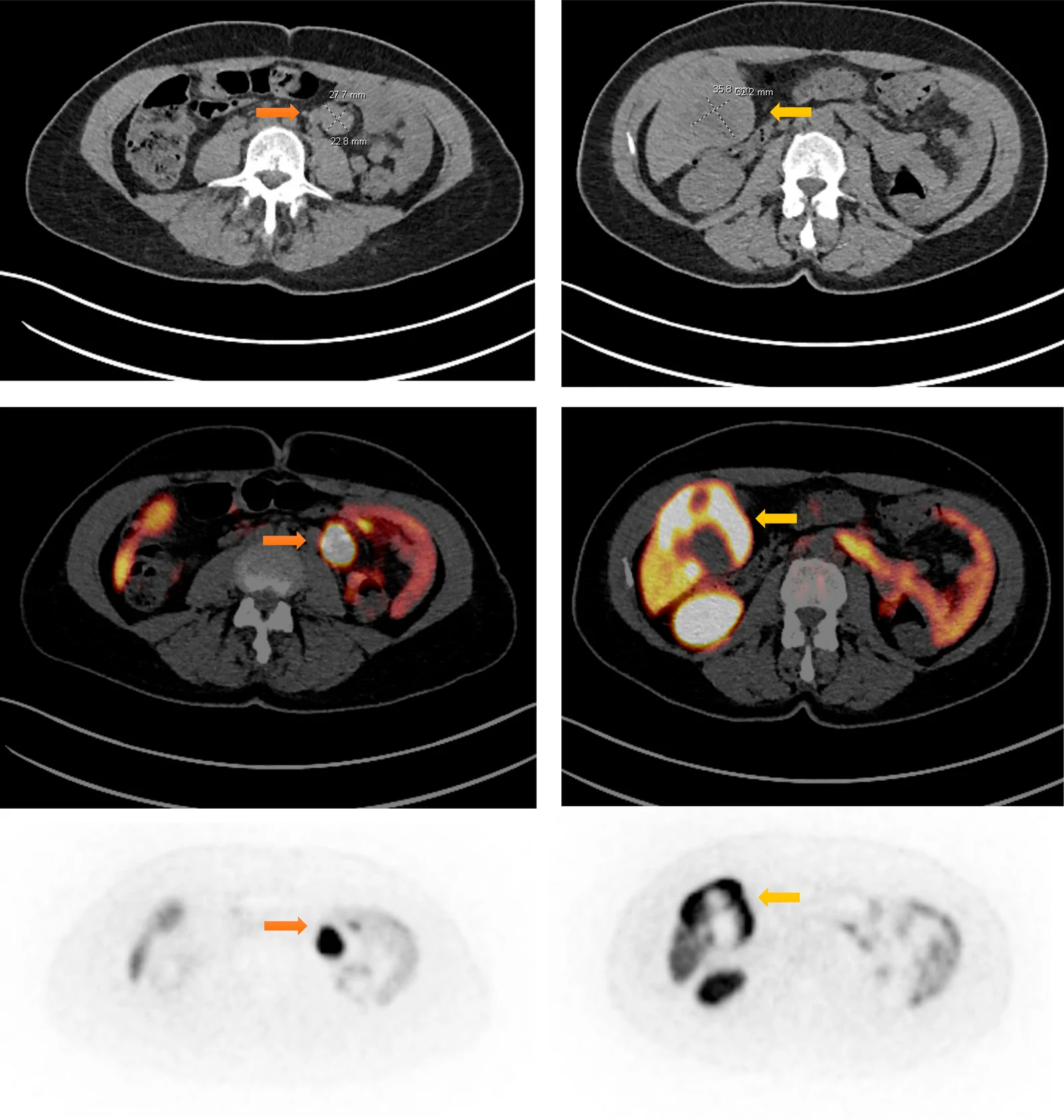
Findings on Gallium - 68 DOTATATE Positron Emission Tomography/Computed Tomography Gallium-68 DOTATATE positron emission tomography/computed tomography scan showing soft tissue mass in the left mid abdomen (red arrow) and ill-defined hypodensity in hepatic segment V (yellow arrow).

**Figure 7:**
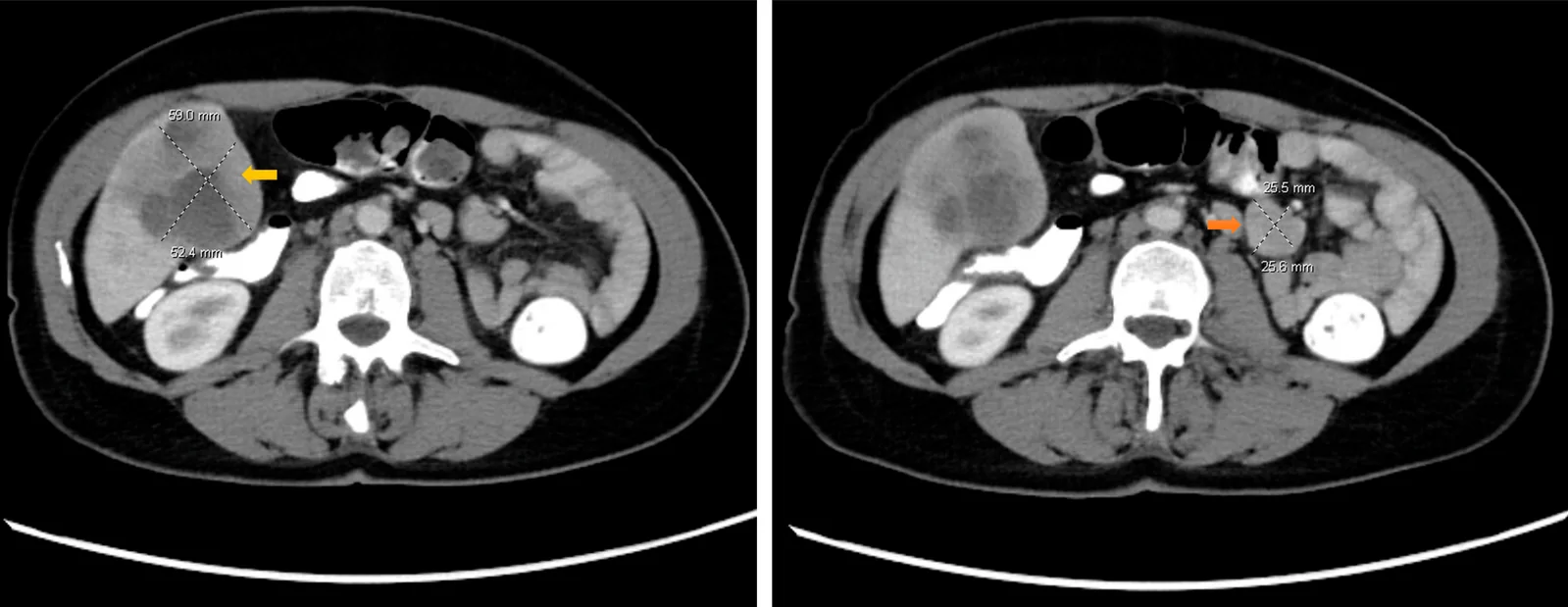
Findings on Computed Tomography of Abdomen Computed tomography of abdomen demonstrates a necrotic mass in the inferior right hepatic lobe measuring approximately 5.2 × 6.0 cm (yellow arrow) and a mesenteric mass in the left hemiabdomen measuring approximately 2.6 × 2.6 cm (red arrow) suspicious for metastatic neuroendocrine disease.

**Figure 8:**
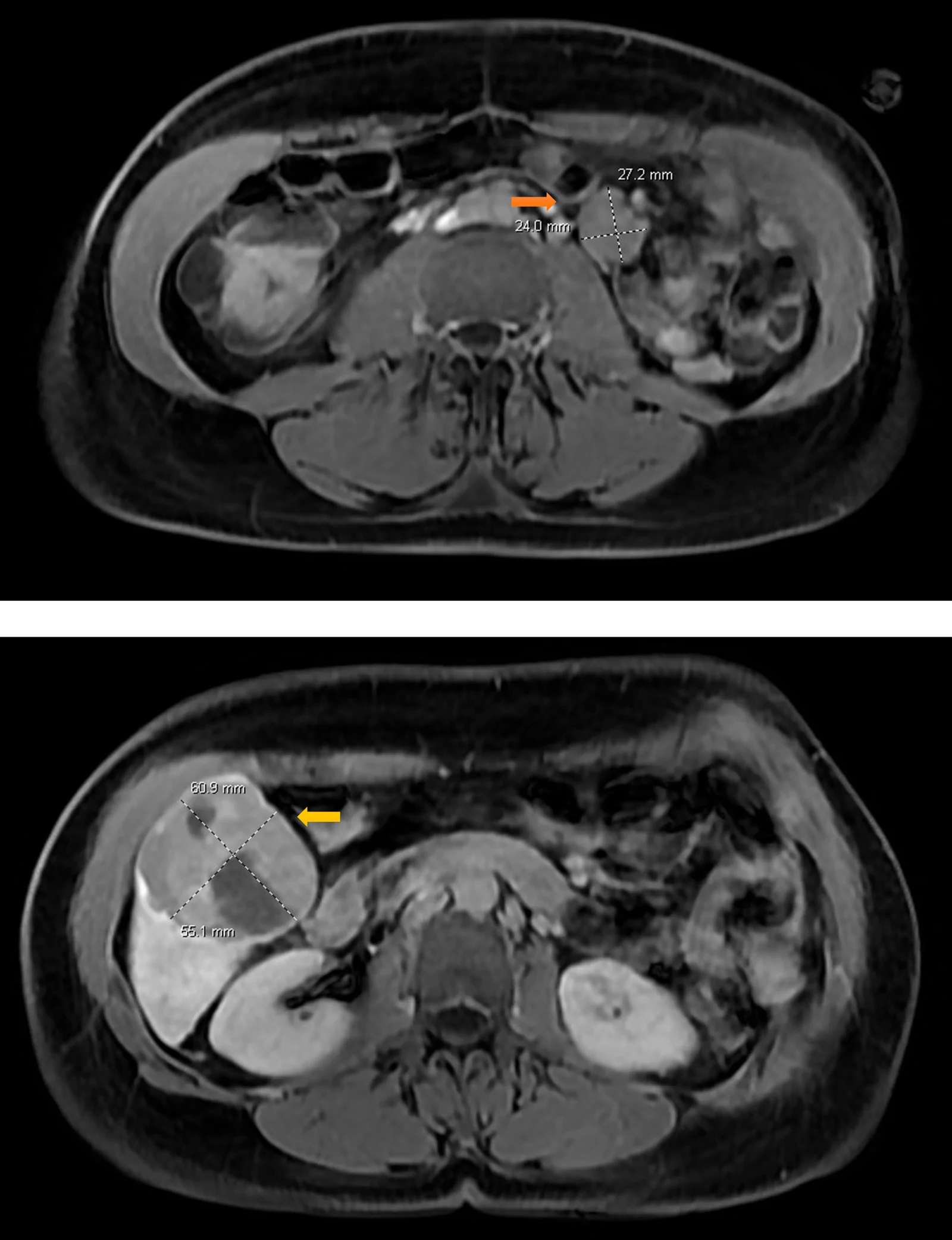
Findings on Magnetic Resonance Imaging of Abdomen Magnetic resonance imaging of the liver showing an enhancing mesenteric mass in the left hemi abdomen measuring approximately 2.7 × 2.4 cm (red arrow) and a heterogeneously enhancing mass (yellow arrow) arising from the hepatic segment 5 extending into the adjacent mesentery abutting the ventral abdominal wall measuring approximately 5.5 × 6.1 cm. The hepatic mass demonstrates focal areas of variable intrinsic T1 and T2 hyperintensity, suggesting cystic and hemorrhagic/proteinaceous contents.

Postoperative biochemical evaluation revealed markedly elevated 24-hour urinary 5-hydroxyindoleacetic acid (5-HIAA) (54.3 mg/24 h; reference ≤7.8 mg/24 h) and chromogranin A (382 ng/mL; reference <93 ng/mL) with normal serum serotonin, consistent with carcinoid syndrome. Gallium-68 DOTATATE positron emission tomography/CT identified a somatostatin receptor-avid mesenteric mass with an adjacent hypermetabolic lymph node and a peripherally hypermetabolic hepatic lesion with central necrosis (Figure 6). Contrast-enhanced CT (Figure 7) and liver magnetic resonance imaging confirmed a 2.6 × 2.6-cm mesenteric mass and a 5.2 × 6.0-cm necrotic hepatic mass in the inferior right lobe, consistent with unilobar metastasis (Figure 8).

Somatostatin analog (SSA) therapy was initiated as a bridge to definitive surgical resection. The patient subsequently underwent exploratory laparotomy with small bowel resection and side-to-side primary anastomosis, resection of a mesenteric mass >5 cm, cholecystectomy en bloc with the hepatic lesion, and partial hepatic lobectomy of segment V. SSA therapy was discontinued following surgical resection, as the patient achieved no evidence of biochemical or radiologic disease.

Final pathology confirmed a unifocal grade 1, well-differentiated small intestinal neuroendocrine tumor (NET) (Ki-67 <3%) measuring 0.9 cm with muscularis propria invasion, lymphovascular invasion, and involvement of a large mesenteric mass; 5 regional and 1 additional mesenteric lymph node were negative. The hepatic specimen showed a single 6.0-cm metastatic grade 1 NET deposit (Ki-67 1%). The gallbladder showed chronic cholecystitis. Final staging was pT2, pN2, pM1a (American Joint Committee on Cancer, ninth edition).

## Outcome and Follow-Up

The patient had an uncomplicated postoperative course following both the cardiac and abdominal surgical procedures. At her 6-week cardiology follow-up, she reported marked improvement in dyspnea and functional capacity. Repeat echocardiography demonstrated well-seated bioprosthetic valves without significant regurgitation or stenosis, normalization of biatrial size, and resolution of right ventricular volume overload. The pulmonary valve was not well visualized; mild residual PR was noted. Biochemical reassessment was planned for 3 months. Cross-sectional imaging was scheduled at 3 months to assess for residual or recurrent disease. She was enrolled in a multidisciplinary NET clinic for coordinated cardio-oncologic surveillance.

## Discussion

Carcinoid heart disease, also known as Hedinger syndrome, arises from serotonin and vasoactive substances secreted by metastatic NETs. Because pulmonary monoamine oxidase inactivates circulating serotonin before it reaches the left heart, CHD shows a near-universal predilection for the tricuspid and pulmonary valves.^1^^,^^2^

Quadrivalvular involvement is rare and recognized through 3 mechanisms: right-to-left shunting through a PFO allowing serotonin-rich venous blood to bypass pulmonary inactivation, saturation of pulmonary monoamine oxidase capacity by massive hepatic tumor burden, and direct left-heart delivery via pulmonary venous drainage from bronchopulmonary carcinoids. The first mechanism is most common, and expert consensus recommends evaluating for a PFO whenever left-sided lesions or unexplained hypoxemia accompany carcinoid syndrome, with shunt closure pursued to prevent ongoing valvular injury.^3^, ^4^, ^5^

A particularly instructive aspect of this case is that the etiology of multivalvular regurgitation was unknown at the time of cardiac surgery, carcinoid valvulopathy had not been considered, and no biochemical evaluation had been performed. Consequently, prophylactic octreotide infusion, standard practice when CHD is recognized preoperatively to prevent intraoperative carcinoid crisis, was not administered. The patient experienced no hemodynamic instability, and her postoperative course was uncomplicated. Current guidelines support echocardiographic screening for CHD when 24-hour urinary 5-HIAA exceeds approximately 300 μmol/24 h (∼50 mg/24 h), a threshold associated with substantially elevated risk of valvular involvement.^6^ Our patient's postoperative 5-HIAA of 54.3 mg/24 h met this threshold precisely, yet the diagnosis followed rather than prompted cardiac evaluation. It was florid quadrivalvular heart failure that drove surgical intervention, and histopathologic analysis of the excised valves that ultimately unmasked the underlying NET, triggering biochemical and tumor workup only in the postoperative period. This reverse-discovery sequence has a practical implication: unexplained multivalvular regurgitation with leaflet thickening and retraction should prompt biochemical screening for carcinoid syndrome regardless of whether an NET diagnosis has been established.

Once CHD was confirmed, postoperative evaluation efficiently established the NET diagnosis. Per 2025 American Society of Clinical Oncology guidelines, SSA therapy is first-line management for carcinoid syndrome, providing symptom control in approximately 70% of patients.^7^ Here, SSA served as a bridge to definitive resection; following complete resection with no evidence of residual disease, SSA was appropriately discontinued.^7^ For patients with progression on SSA, peptide receptor radionuclide therapy with lutetium-177 dotatate is preferred for somatostatin receptor-positive tumors, with everolimus as an alternative for receptor-negative disease.^8^ Cabozantinib, evaluated in the CABINET (Randomized, Double-Blinded Phase III Study of Cabozantinib Versus Placebo in Patients With Advanced Neuroendocrine Tumors After Progression on Prior Therapy) trial, is an emerging option for previously treated progressive NETs.^9^ Cholecystectomy was performed concomitantly given the risk of SSA-induced cholelithiasis.^7^ Surgical resection of the primary tumor and accessible metastases should be pursued when technically feasible,^7^ and published series confirm that quadrivalvular carcinoid involvement can be managed surgically with acceptable medium-term outcomes in selected patients.^3^^,^^4^^,^^10^ Postsurgical oncologic surveillance should include multiphasic CT or magnetic resonance imaging of the abdomen and pelvis every 3 to 12 months initially, transitioning to every 12 to 24 months for up to 10 years.^8^

## Conclusions

CHD is classically a right-sided valvular disorder, but quadrivalvular involvement represents a clinically distinct and challenging subset. This case highlights the indolent course and nonspecific symptoms that can delay diagnosis. Atypical multivalvular involvement should prompt systematic biochemical screening for carcinoid syndrome. Preoperative recognition is critical; failure to identify CHD before surgery exposes patients to the risk of intraoperative carcinoid crisis, and prophylactic octreotide infusion must be administered when the diagnosis is established or suspected before any surgical intervention.

**Take-Home Messages**

• Unexplained multivalvular regurgitation with retracted, thickened leaflets should raise the suspicion for carcinoid syndrome even without a known NET diagnosis.
• CHD is typically right-sided; quadrivalvular involvement should prompt evaluation for a PFO or other mechanism bypassing pulmonary metabolism, and when identified, shunt closure should be pursued to prevent ongoing valvular injury.
• Multivalve replacement, shunt closure, and tumor-directed therapy should be coordinated at a higher-volume NET specialty center, with cross-sectional imaging surveillance maintained for up to 10 years.

## Funding Support and Author Disclosures

This case study received no specific grant from any funding agency in the public, commercial, or not-for-profit sectors. The authors have reported that they have no relationships relevant to the contents of this paper to disclose.

## Appendix

Video 1 TTE Zoomed in PLAX View of the Mitral Valve Showing a Thickened Mitral Valve With Anterior Leaflet Prolapse and Severe Posteriorly Directed Mitral Regurgitation PLAX = parasternal long axis view.

Video 2 TEE Mid Esophageal View at 0° Showing Thickened, Restricted, and Malcoapted Tricuspid Valve With Resultant Severe Tricuspid Regurgitation

Video 3 TEE Midesophageal View at 0 and 120° Showing a Thickened, Restricted Mitral Valve, Flail Anterior Leaflet With Severe Posteriorly Directed Mitral Regurgitation

Video 4 TEE Midesophageal View Zoomed in Long Axis view at 110° Showing Severe Aortic Regurgitation

Video 5 TEE Midesophageal Short Axis View at 45° Showing Moderate Pulmonary Regurgitation

Video 6 TEE Bicaval View With a large PFO and Bidirectional Shunt on Color Doppler
